# Probiotics in Infancy and Childhood for Food Allergy Prevention and Treatment

**DOI:** 10.3390/nu16020297

**Published:** 2024-01-18

**Authors:** Margherita Di Costanzo, Adriana Vella, Claudia Infantino, Riccardo Morini, Simone Bruni, Susanna Esposito, Giacomo Biasucci

**Affiliations:** 1 Pediatrics and Neonatology Unit, Guglielmo da Saliceto Hospital, 29121 Piacenza, Italy; giacomo.biasucci@unipr.it; 2 Department of Medicine and Surgery, University of Parma, 43126 Parma, Italy; 3 Pediatric Clinic, Department of Medicine and Surgery, University of Parma, 43126 Parma, Italy; adrivella.95@gmail.com (A.V.); infantinoclaudia@gmail.com (C.I.); riccardomorini95@gmail.com (R.M.); simone.bruni@unipr.it (S.B.); susannamariaroberta.esposito@unipr.it (S.E.)

**Keywords:** oral tolerance, gut microbiota, gut dysbiosis, cow’s milk allergy, egg allergy, shellfish allergy, food specific immunotherapy

## Abstract

Food allergy represents a failure of oral tolerance mechanisms to dietary antigens. Over the past few years, food allergies have become a growing public health problem worldwide. Gut microbiota is believed to have a significant impact on oral tolerance to food antigens and in initiation and maintenance of food allergies. Therefore, probiotics have also been proposed in this field as a possible strategy for modulating both the gut microbiota and the immune system. In recent years, results from preclinical and clinical studies suggest a promising role for probiotics in food allergy prevention and treatment. However, future studies are needed to better understand the mechanisms of action of probiotics in food allergies and to design comparable study protocols using specific probiotic strains, defined doses and exposure times, and longer follow-up periods.

## 1. Introduction

Food allergy represents a major health issue in Western countries due to its increasing prevalence in the last several decades, reaching rates of 8% in children and 3% in adults [1]. In the last few years, an increase in the severity of food-induced allergic reactions, such as anaphylaxis, has been reported in children [2]. Allergens responsible for allergic reactions are usually different in children and adults; indeed, peanut (2.2%), milk (1.9%), shellfish (1.3%), and tree nuts (1.2%) are the most common allergens in children, whereas shellfish (2.9%), milk (1.9%), peanut (1.8%), tree nuts (1.2%), and finfish (0.9%) are the most common ones in adults [3]. Furthermore, different allergens generally result in food allergies with a different clinical course. Thus, childhood food allergies to cow’s milk, egg, wheat, or soy typically resolve during childhood, while food allergies to peanuts, tree nuts, fish, and shellfish are usually persistent in adulthood. However, a changing pattern in food allergies has been observed in the last thirty years, with an increased risk of persistence up to later ages [3,4]. Beside this, it is well known that individuals with food allergies are at a higher risk of developing other allergic conditions later in life as part of the atopic march. These conditions may include allergic rhinitis, conjunctivitis, and asthma [5]. Recent epidemiological changes have placed a significant burden on patients, their caregivers, and healthcare systems. Therefore, it is very important to develop effective strategies for the management of food allergies, starting at a young age. The elimination diet is currently the first-line treatment for all children with food allergies. There is increasing evidence that the gut microbiota plays a crucial role in overall health. It is likely that gut dysbiosis, which is an imbalance in gut microbiota composition and functions, anticipates the development of food allergies [6]. Thus, modulation of gut microbiota has become a potential tool for prevention and treatment of food allergies. Hence, the use of probiotics has been claimed as one of the possible strategies to modulate gut microbiota composition and functions.

This paper aims to review the most recent and relevant preclinical and clinical studies on the use of probiotics in the management of food allergies, analyzing the real possibility of an effective strategy for their prevention and treatment in the future.

## 2. Gut Dysbiosis and Food Allergies

Based on the hypothesis that gut dysbiosis may play a role in the development of food allergies, many studies have been carried out to assess whether gut microbiota composition and functions could be associated with the development, persistence, or resolution of food allergies, as well as which biological mechanisms could be involved.

Several observational studies have shown that gut dysbiosis plays a role in the development of food allergies [6,7,8,9,10,11,12,13,14,15]. The currently available studies are quite diverse, and no specific bacterial group has been definitively linked to the onset or clinical course of food allergy [6,7,8,9,10,11,12,13,14,15]. However, the results of observational studies suggest that gut dysbiosis may precede the onset of food allergies. Additionally, research indicates that gut microbiota during early life, particularly in the first 6 months of life, play a crucial role not only in the development but also in the persistence of food allergies until adulthood [10]. A recent study analyzed the fecal microbiome and metabolome of food allergy concordant or discordant twin pairs, suggesting a potential role of gut dysbiosis in food allergies beyond infancy and into adulthood. Data analysis revealed a diverse gut microbiota and metabolites in twins with food allergies compared to healthy twins, even within the same twin pairs, both in infancy and adulthood. These findings suggest that the gut microbiota may have a protective role against food allergies, even in adulthood [16].

Further studies suggested that *Akkermansia muciniphila* and *Faecalibacterium prausnitzii* contribute to allergic diseases and that their colonization in the gut microbiota is modified in atopic patients compared to healthy controls [17,18,19]. However, current studies in pediatric patients are preliminary and only focused on atopic dermatitis and allergic asthma [17,18,19].

In a cross-sectional observational pilot study, Fieten et al. analyzed the fecal microbiome of children with atopic dermatitis with or without a concomitant food allergy and found that *F. prausnitzii* and *A. muciniphila* discriminate between the presence and absence of food allergy in children with atopic dermatitis (*p* = 0.001). The fecal microbiome of children with atopic dermatitis and food allergies harbored relatively less *F. prausnitzii* and *A. muciniphila* than that of children with atopic dermatitis without food allergies [20]. 

De Filippis et al. identified specific microbial signatures in the gut microbiome of allergic children affected by food or respiratory allergies, such as a higher abundance of *Ruminococcus gnavus* and *F. prausnitzii* and a depletion of *Bifidobacterium longum, Bacteroides dorei, B. vulgatus*, and fiber-degrading taxa [21]. The authors hypothesized that the increased abundance of *F. prausnitzii* reported in allergic subjects in this and previous studies was probably linked to an increase in *F. prausnitzii* clade A, previously associated with the Westernized lifestyle [22]. Interestingly, Song et al. found an increase in *F. prausnitzii* strain L2-6 (belonging to clade A) in atopic dermatitis, suggesting a role of this *F. prausnitzii* clade in allergy development [23].

Observational studies in humans, however, provide no evidence about a causal relationship between gut dysbiosis and the development of food allergies and do not elucidate the mechanisms involved. Animal models show that antibiotic-treated mice exhibit a predisposition to allergy development, while germ-free mice do not develop oral tolerance but maintain a Th2 immune response to oral administration of food antigens [24,25]. This condition may only be reversed by early gut microbiota remodulation. These data support the pivotal role of gut microbiota in establishing oral tolerance to dietary antigens early in life. Indeed, germ-free mice, colonized with feces from healthy donors, are protected from developing cow’s milk allergy (CMA) upon sensitization and challenge to cow’s milk proteins. In contrast, germ-free mice colonized with feces from infants with CMA exhibit severe allergic responses, including anaphylaxis [26].

There are multiple mechanisms by which the gut microbiota may influence food allergy predisposition. Murine models of food allergy have shown several effects of the gut microbiota, including modulation of the Th2 immune response, regulation of the development of mucosal immunity and oral tolerance, regulation of basophil populations, and promotion of gut barrier function through reduced gut permeability and increased mucus production [27].

Also, even metabolites resulting from the gut microbiota functions have an emerging role in food allergies. Butyrate is a short-chain fatty acid produced by the fermentation of dietary fiber in the colon. It has a strong immunoregulatory effect, which is expressed through both immune and non-immune mechanisms of action. Indeed, butyrate can improve the integrity of the intestinal epithelial barrier by increasing the thickness of the mucus layer and the expression of tight junctions. Alongside these effects, it has several direct and indirect effects on immune cells that contribute to the induction and maintenance of oral tolerance [28].

As for butyrate production, a Canadian longitudinal study showed that infants who develop allergic sensitization do not differ from those who already have gut dysbiosis with reduced butyrate production at 3 months of age [29].

On the other hand, the protective effects of breastfeeding on food allergies may be partly explained by the human milk butyrate content, which has been demonstrated to modulate several tolerogenic mechanisms. Human milk butyrate could at least partly explain the breastfeeding protective effect towards food allergies. This effect has been tested in animal models *in vivo* and in cellular models *in vitro*. Butyrate can regulate gut barrier function, promote the activation of regulatory T cells (Tregs), and modulate the Th1/Th2 response in favor of a tolerogenic Th1 immune response [30].

## 3. Probiotic-Induced Gut Microbiota Modulation for Food Allergy Prevention and Treatment

### 3.1. Probiotics and Their Mechanisms of Action

According to the widely recognized FAO/WHO definition [31], revised in a consensus statement by the International Scientific Association for Probiotics and Prebiotics [32], probiotics are defined as “live microorganisms that, when administered in adequate amounts, confer a health benefit on the host”. To meet this definition, probiotics must be present in a reasonable amount within the product. It has been suggested that at least 1 × 10^9^ colony-forming units (CFU) are required to ensure gut colonization and exert measurable beneficial effects [33]. Lower amounts can be used in cases where robust scientific evidence supports the specific strain’s colonization ability. Probiotics are available on the market in many different forms, including medicinal products, medical devices, dietary supplements, and foodstuffs. Globally, the probiotics market has steadily grown in the last few years. 

This is true for all probiotic formulations available and at any age, both in childhood and adulthood [34]. Considering this increase in marketing, it is worthy of being emphasized that not all probiotics are the same and/or provide the same beneficial effects. Growing evidence shows that the efficacy of probiotics is strain- and disease-specific [35]. Therefore, health professionals should take these two aspects into account when recommending probiotics. 

Different strains of probiotics have distinct mechanisms of action. This may explain why some probiotics, unlike others, are effective against specific diseases and conditions [35]. Current preclinical and clinical data regarding the possible role for probiotics in the prevention and treatment of allergies, specifically food allergies, are encouraging but not yet sufficient to strongly recommend the use of probiotics in these conditions. In the following sections, we will discuss the main preclinical and clinical studies that have evaluated the role of probiotics and their mechanisms of action in the prevention and treatment of pediatric food allergies.

### 3.2. Data from Animal Models of Food Allergy

Mice are the most widely used animal models to study the pathophysiological mechanisms underlying IgE-mediated food allergies, and the possible preventive and therapeutic strategies for cow’s milk, egg, and shellfish allergies. Herein, we report the most recent studies, limited to the last five years, focused on the role of specific probiotic strains in mouse models of food allergies.

#### 3.2.1. Cow’s Milk Allergy

In a recent study, *Lactobacillus* (L.) *plantarum* HM-22 has been administered by gavage to α-lactalbumin-induced allergic mice for five weeks to investigate its possible effect on gut inflammation and microbiota. The study demonstrated that *L. plantarum* HM-22 induced a significant increase in serum levels of tolerogenic cytokines, including IL-10, IFN-γ, and TGF-β, and a significant decrease in serum total IgE and IL-4 levels in mice with α-lactalbumin-induced allergy. The colonic tissue crypt structure of α-lactalbumin-induced allergic mice was initially altered, resulting in reduced goblet cells and increased inflammatory corpuscles, but *L. plantarum* HM-22 administration was found to attenuate these effects. Furthermore, *L. plantarum* HM-22 significantly increased the expression of occludin and claudin-1 in the colon of α-lactalbumin-induced allergic mice, thereby reducing gut permeability. In addition, *L. plantarum* HM-22 enhanced gut microbiota colonization in α-lactalbumin-induced allergic mice [36].

*Lactobacillus acidophilus* KLDS 1.0738 was found to alleviate β-lactoglobulin-induced allergic inflammation in a mouse model of cow’s milk allergy (CMA) [37]. Furthermore, Li et al. investigated its molecular regulation mechanism in β-lactoglobulin-induced macrophages, treated with viable or non-viable strains of *L. acidophilus* KLDS 1.0738 and Toll-like receptor 4 (TLR4) inhibitors or miR-146a inhibitors. The results showed that treatment with *L. acidophilus* KLDS 1.0738 may suppress the TLR4/NF-κB signaling pathway by modulating miR-146a expression, thereby reducing the overexpression of downstream inflammatory factors [38].

Fu et al. assessed the impact of three *Lactobacillus* strains on the immune system, gut barrier, and gut microbiota in β-lactoglobulin-induced allergic mouse model. Oral administration of *L. plantarum* ZDY2013 and *L. rhamnosus* GG (LGG) suppressed the allergic response by reducing serum total IgE levels, attenuating anaphylaxis symptoms, and inducing Th1 immune cells or Tregs differentiation to inhibit the Th2 immune response. In addition, *L. plantarum* ZDY2013 and LGG improved gut barrier function through tight junction regulation, and *L. plantarum* ZDY2013 and *L. plantarum* WLPL04 regulated gut dysbiosis in allergic mice [39].

#### 3.2.2. Egg Allergy

In a recent study, the use of *Bifidobacterium longum* subsp. longum 51A (BL51A) was evaluated in a mouse model of ovalbumin (OVA) food allergy. BL51A was orally administered and resulted in a reduction of OVA-specific serum IgE levels, gut permeability, proximal jejunal damage, eosinophil and neutrophil recruitment, and levels of eotaxin-1, CXCL1/KC, IL-4, IL-5, IL-6, IL-13, and TNF. In addition, this treatment increased IL-10 levels [40]. Recently, similar results were obtained for *Akkermansia muciniphila* BAA-835 in a mouse model of OVA food allergy [41]. However, a recent study found that the impact of *A. muciniphila* is context-dependent and can be detrimental to food allergies when the microbiota is deprived of dietary fiber. To investigate the causal role of *A. muciniphila* in modulating food allergies, the authors used germ-free mice colonized with a fully characterized 14-member synthetic human gut microbiota, in which *A. muciniphila* can be included or excluded. The study found that the presence of *A. muciniphila* in the microbiota, combined with fiber deprivation, led to stronger anti-commensal IgE coating and innate type 2 immune responses. This worsened food allergy symptoms in animal models of OVA and peanut allergy [42].

Duan et al. investigated the effects of oral administration of *L. plantarum* JC7 using a mouse model of OVA sensitization. The authors showed that *L. plantarum* JC7 significantly alleviated allergic manifestations; it also reduced plasma histamine levels, OVA-specific serum IgE levels, and shifted Th1/Th2 and Treg/Th17 imbalances. This was achieved by promoting the secretion of IL-10 and IFN-γ tolerogenic cytokines, meantime, inhibiting secretion of those involved in the allergic response, such as IL-4 and Th17. The observed effects may be attributed to the activation of the NF-κB signaling pathway. In addition, OVA-sensitized group showed gut dysbiosis that was restored by *L. plantarum* JC7 oral administration. Specifically, oral administration of *L. plantarum* JC7 increased the richness, diversity, and uniformity of cecum microbiota, which was characterized by a higher abundance of Bacteroidetes and reduced Firmicutes colonization [43]. 

In another study by Miranda et al., the probiotic effect of *Saccharomyces cerevisiae* UFMG A-905 was evaluated in an OVA food allergy model. The authors also evaluated if *Saccharomyces cerevisiae* UFMG A-905 might be effective after inactivation. The study found that oral administration of only viable probiotics significantly reduced tissue damage and myeloperoxidase activity, as well as IL-17 levels. However, this study did not find any significant changes in the serum OVA-specific IgE and IgG levels. This suggests that the observed effects in the evaluated murine model were local rather than systemic [44].

#### 3.2.3. Shellfish Allergy

Fu et al. demonstrated different effects of oral administration of five distinct strains of lactic acid bacteria in alleviating gut allergic inflammation and symptoms related to food-induced anaphylaxis in a mouse model of food allergy to shrimp tropomyosin, a major shrimp allergen. The most effective strain in reducing allergies was *Bacillus coagulans* 09.712, which significantly improved epithelial barrier function and increased lymphocyte proliferation. *Bacillus coagulans* 09.712 induces CD4+Foxp3+Tregs production, which suppresses the pro-inflammatory Th17 response in this allergic mouse model. Also, *Bacillus coagulans* 09.712 administration suppresses mTOR activation, resulting in up-regulation of FOXP3 and down-regulation of GATA-3, which, in turn, facilitates the control of tropomyosin-induced pro-inflammatory Th2 and Th17 immune responses [45].

Oral administration of *L. casei* Zhang probiotic strain reduced allergy symptoms and gut epithelial damage in a mouse model of tropomyosin-induced food allergy. In addition, administration of *L. casei* Zhang changed development and function of dendritic cells (DCs), T cells, and B cells, resulting in a tropomyosin-specific antibody isotypes shift towards a more tolerogenic pattern through the activation of the NF-κB signaling pathway [46]. Moreover, in a previous study, the same authors demonstrated that even *Bifidobacterium (B.) infantis* can alleviate shrimp tropomyosin-induced food allergy in mice by tolerogenic DCs-dependent Treg induction, by and a favorable gut microbiota modulation [47] (Table 1).

In conclusion, though the results of probiotics use in animal models of food allergies look promising, they cannot be immediately translated to humans due to many genetic and environmental factors that may influence food allergies onset and course. Nonetheless, well-designed animal models may be useful for future studies to better understand the mechanisms that underlie specific probiotic strains effects in food allergies.

### 3.3. Data from Human Studies Related to Food Allergy Prevention

Clinical studies that have evaluated the role of probiotics in the prevention of food allergies are based on three different approaches:

- administration of probiotics only to the mother during pregnancy and breastfeeding;
- administration of probiotics to mother and infant in the perinatal period;
- administration of probiotics only to infants after delivery.

#### 3.3.1. Administration of Probiotics Only to the Mother during Pregnancy and Breastfeeding

Boyle et al. conducted a randomized controlled trial to investigate the effects of prenatal treatment with LGG on pregnant mothers from 36 weeks of gestation until delivery. The study found that this treatment did not reduce the risk of eczema and food sensitization to eggs, peanuts, and cow’s milk in infants at high risk of developing allergic diseases, based on a one-year follow-up [48].

In a double-blind, randomized trial, pregnant women were given either probiotic-supplemented milk or placebo from 36 weeks of gestation until three months postpartum while breastfeeding. The probiotic milk contained LGG, *L. acidophilus* La-5, and *B. animalis* subsp. *lactis* Bb-12. At two years of age, their children underwent assessments for atopic sensitization, atopic dermatitis, asthma, and allergic rhino-conjunctivitis. The authors concluded that administering probiotics to non-selected mothers reduced atopic dermatitis overall incidence, but did not have any impact on atopic sensitization. The tested trophoallergens included cow’s milk, hen egg white, cod, hazelnut, and peanut [49].

Rautava et al. conducted a double-blind, placebo-controlled study to determine if probiotic supplementation during pregnancy and breastfeeding in mothers with allergic diseases and atopic sensitization could reduce the risk of eczema development in infants, with a two-year follow-up. Mothers were randomized to receive: *L. rhamnosus* LPR and *B. longum* BL999; *L. paracasei* ST11 and *B. longum* BL999; or placebo, starting two months before delivery throughout the first two months after delivery, while breastfeeding. Infants of mothers who received *L. rhamnosus* LPR and *B. longum* BL999 or *L. paracasei* ST11 and *B. longum* BL999 had a significantly lower risk of developing eczema during the first two years of life compared to placebo. In contrast, the two probiotic mixtures did not affect the risk of infants’ atopic sensitization. The tested trophoallergens included cow’s milk, hen egg white, wheat and rice flour, cod, soybean, potato, carrot, and banana [50]. 

#### 3.3.2. Administration of Probiotics to Mother and Infant in the Perinatal Period

One of the first large sample size studies was a double-blind, placebo-controlled trial conducted in mothers with infants at high risk of allergy. Pregnant women were randomized to receive a probiotic mixture consisting of two lactobacilli, bifidobacteria, and propionibacteria (a capsule containing freeze-dried LGG, *L. rhamnosus* LC705, *B. breve* Bb99, and *Propionibacterium freudenreichii* ssp. *shermanii* JS) or placebo during the last month of pregnancy, and their infants were to receive it from birth until age 6 months. Infants also received a prebiotic galactooligosaccharide or placebo. At five years, the cumulative incidence of allergic diseases (eczema, food allergy, allergic rhinitis, and asthma) and IgE sensitization did not differ between the two study groups. However, there were fewer IgE-associated allergic diseases in cesarean section delivered children who received probiotics. The authors concluded that probiotic supplementation during the last month of pregnancy, and during the first six months of infants’ life, is not effective in reducing the incidence of allergic diseases at five years of age [51]. 

Indeed, in a previous double-blind, randomized, placebo-controlled study, a single LGG probiotic strain was administered prenatally to mothers with infants at high risk allergy and postnatally to their infants for six months. The authors found that LGG was effective in preventing early atopic disease in high-risk children throughout a two-year follow-up period [52].

#### 3.3.3. Administration of Probiotics Only to Infants after Delivery

Postnatal administration of a probiotic mixture consisting of *B. infantis*, *B. lactis*, and *Streptococcus thermophilus* did not affect the incidence of allergic manifestations or atopic sensitization during the first two years of life in very preterm newborns [53]. In this study, food allergy was defined according to a parental report of a physician-diagnosed allergy to cow’s milk, soy, egg, wheat, or peanut. Skin prick tests were performed only for egg white, cow’s milk, and peanut. Additionally, this study did not evaluate the strain- and disease-specific probiotic effect. 

In a multicenter, randomized, double-blind, controlled study, a non-hydrolyzed fermented infant formula containing heat-killed *B. breve* C50 and *Streptococcus thermophilus* 065 (HKBBST) was administered to infants at high risk of atopy during their first year of life. The use of HKBBST milk did not affect the proportion of CMA but reduced the proportion of positive skin prick tests to cow’s milk and the occurrence of allergy-like events in the first two years of life [54] (Table 2).

In 2015, the World Allergy Organization (WAO) issued guidelines on probiotics for the prevention of allergic diseases. The WAO guideline panel suggests supplementation with probiotics in pregnant women at high risk of having an allergic child, in women who breastfeed infants at high risk of developing allergies, and in infants at high risk of developing allergies. The authors specified that all recommendations are conditional and supported by very low-quality evidence [55].

In 2016, Zhang et al. conducted a PRISMA-compliant systematic review and meta-analysis of randomized controlled trials on probiotics for the prevention of atopy and food hypersensitivity in early childhood. The results indicated that administering probiotics prenatally and postnatally could reduce the risk of atopy and food hypersensitivity in young children [56]. 

To date, many studies have evaluated the role of probiotics in preventing food sensitization without assessing their effects on confirmed food allergy prevention. Therefore, further studies are needed to determine the effectiveness of probiotics as a global prevention strategy for food allergies. Future studies should also assess the optimal probiotic strains, dosing, and duration of therapy and should be designed with a long-term follow-up period.

### 3.4. Data from Human Studies Related to Food Allergy Treatment

Most of the available clinical studies on the use of probiotics as a possible therapeutic strategy for pediatric food allergies focus on IgE-mediated CMA, which is the earliest and most common food allergy in pediatrics [57]. It is usually resolved at school age, but the natural history of food allergies has changed in recent years, and persistent forms of food allergy in adulthood are increasingly common [47]. Currently, CMA therapy is based on cow’s milk proteins elimination diet replaced by the use of special alternative formulas in non-breastfed infants [58]. Special formulas mostly used for the management of CMA are: extensively hydrolyzed whey formula (eHWF), extensively hydrolyzed casein formula (eHCF), soy formula (SF), hydrolyzed rice formula (HRF), and amino acid-based formula (AAF) [59]. These hypoallergenic formulas resolve allergic symptoms by lacking IgE-binding epitopes [60]. However, besides ameliorating allergic symptoms, it should be crucial to find strategies to promote oral tolerance in patients with food allergies.

Berni Canani et al. demonstrated that in children with IgE-mediated CMA, LGG-supplemented eHCF resulted in higher rates of oral tolerance compared to eHCF without LGG and other hypoallergenic formulas used in CMA treatment [61].

These findings were consistent with those of a one-year follow-up study conducted in the United States, which showed better outcomes using eHCF plus LGG compared to eHCF alone or AAF, as first-line CMA dietary management in infants [62].

The use of eHCF plus LGG for the treatment of IgE-mediated CMA in children is associated with a higher rate of oral tolerance acquisition and a lower incidence of atopic manifestations compared to the use of eHCF alone, or other special formulas for CMA treatment (e.g., HRF, SF, eHWF, AAF), even after a 36-months follow-up [63,64]. These results align with those of a retrospective study performed in the United Kingdom based on a large cohort of formula-fed CMA infants extracted from the Health Improvement Network database, which indicated that eHCF plus LGG is not only more effective than eHWF in managing CMA symptoms, but it also has greater potential to prevent the occurrence of other atopic manifestations in these patients [65].

Basturk et al. conducted a randomized, double-blind, placebo-controlled trial in CMA infants who received oral LGG for 4 weeks. The mothers of all breastfed patients were put on a milk-free diet, and all formula-fed patients were offered eHF. The probiotic group showed statistically significant improvement in symptoms such as bloody stools, diarrhea, restiveness, and abdominal distension, as well as improvement in mucous stools and vomiting, compared to the placebo group. In contrast, a statistically significant improvement in abdominal pain, constipation, and dermatitis was not observed. Although the probiotic group had higher complete recovery rates than the placebo group, the difference was not statistically significant [66].

All the clinical studies presented so far have evaluated the role of LGG alone, or in addition to formula, in the management of infants and children with IgE-mediated CMA. However, there is also evidence regarding the role of some *Bifidobacteria* strains in the treatment of CMA.

In a randomized, double-blind, placebo-controlled trial, Jing et al. demonstrated that *B. bifidum* TMC3115 supplementation reduced allergic symptoms, improved anti-inflammatory responses, reduced serum IgE levels, increased serum IgG2 levels, and improved gut microbiota in infants with CMA [67].

Strisciuglio et al. investigated the effect of *Bifidobacteria* on the phenotype and activation status of peripheral basophils and lymphocytes in children with CMA. The treatment with *Bifidobacteria* resulted in a decrease in circulating naive and activated CD4+ T cells, as well as degranulating basophils. The authors concluded that *Bifidobacteria* may have beneficial effects on in modulating oral tolerance in children with CMA [68] (Table 3).

In 2019, Qamer et al. evaluated the use of probiotics for CMA in the first systematic review of randomized controlled trials. The authors concluded that there is limited, low-quality evidence indicating that probiotic supplementation may be associated with earlier acquisition of oral tolerance to cow’s milk proteins in children with CMA. However, the authors specified that large, well-designed trials are necessary to confirm these findings [69].

In 2022, the Global Allergy and Asthma European Network (GA^2^LEN) made no recommendation for or against any probiotics in managing food allergies, whether used as a supplement or added to infant formulas. GA^2^LEN suggested addressing high-quality prospective trials on infants and young children with documented food allergies [70].

### 3.5. Probiotics in Food-Specific Immunotherapy

Oral immunotherapy is one of the possible allergen-specific therapeutic strategies proposed for the management of food allergies. The primary goal of oral immunotherapy is to induce desensitization to the allergen, but it is often burdened by allergic reactions. Probiotics have been evaluated in combination with oral immunotherapy to enhance their effectiveness or mitigate their adverse effects.

In 2015, Tang and colleagues published the first double-blind, placebo-controlled, randomized trial of a combined therapy with a probiotic, *L. rhamnosus* CGMCC 1.3724, and peanut oral immunotherapy in children with peanut allergies. They found that probiotic and peanut oral immunotherapy were highly effective, with seven children achieving possible sustained unresponsiveness if nine were treated [71].

The same study group later demonstrated that combined therapy with *L. rhamnosus* CGMCC 1.3724 and peanut oral immunotherapy provided long-lasting clinical benefit compared to placebo, with two-thirds of treated participants symptom-free after peanut ingestion 4 years after completing treatment [72].

Moreover, the authors described another study protocol of a multicentre, randomized, controlled trial evaluating the effectiveness of probiotic *L. rhamnosus* CGMCC 1.3724 and peanut oral immunotherapy in inducing desensitization or tolerance in children with peanut allergy compared with oral immunotherapy alone and with placebo [73].

In a multicenter, randomized, phase 2b trial, another probiotic, *L. rhamnosus* ATCC 53103, plus peanut oral immunotherapy, was compared to peanut oral immunotherapy plus placebo in children aged 1–10 years with a confirmed diagnosis of peanut allergy through oral challenge. The authors concluded that both treatments were able to induce desensitization, and the addition of the probiotic did not improve treatment efficacy but might offer a safety benefit [74].

Based on the observations in peanut allergy, Loke et al. planned the first double-blind, placebo-controlled, randomized trial to examine the effectiveness of probiotic and egg oral immunotherapy in inducing desensitization or sustained unresponsiveness in children with egg allergy compared to placebo [75].

## 4. Conclusions, Limitations and Future Perspectives

This narrative review outlines the current preclinical and clinical studies on the use of probiotics in the management of food allergies in infancy and childhood, exploring available data from animal models of cow’s milk, egg, and shellfish allergies as well as data from human studies related to food allergy prevention and treatment. The available evidence is not conclusive, but it suggests that probiotics may have a role in preventing and treating food allergies in pediatrics. The lack of consistency is due to the wide range of probiotic strains used in studies based on different study protocols. 

The most promising results available concern the use of specific probiotic strains as adjuvants in the management of children with IgE-mediated cow’s milk allergies, as well as the use of specific probiotic strains in oral immunotherapy for children with IgE-mediated peanut allergies.

Nevertheless, current studies lay the groundwork for future well-designed studies to eventually identify specific probiotic strains that may be effective in the management of food allergies, the relative optimal dose to be administered, and the proper duration and timing of administration. 

It is also critical that these studies do not confuse food allergy with allergic sensitization to food antigens, as this could be an additional confounding factor in interpretation. Indeed, the diagnosis of food allergies should be based on a positive oral food challenge.

Another future goal should be to clarify the molecular mechanisms by which probiotics interact with host cells and the gut microbiota and how these interactions affect the immune response to food antigens, including complex epigenetic mechanisms.

## Figures and Tables

**Table 1:** Data from animal models of food allergies.

Food	Study	Probiotic	Findings
Cow’s milk	Jiang et al. [36]	*L. plantarum* HM-22	Increased serum levels of IL-10, IFN-γ, and TGF-β; Reduced serum levels of total IgE and IL-4; Reduced gut permeability (increased expression of occludin and claudin-1 in the colon).
	Ni et al. Li et al. [37,38]	*L. acidophilus* KLDS 1.0738	Suppression of the TLR4/NF-kB signaling pathway
	Fu et al. [39]	*L. plantarum* ZDY2013 and *L. rhamnosus GG* (LGG)	Reduced serum levels of total IgE; Promoted Th1 differentiation, inhibiting Th2 responses; Improved gut barrier function.
Egg	Santos et al. [40]	*B. longum* 51A	Reduced serum levels of total IgE, gut permeability, proximal jejunal damage, eosinophil and neutrophil recruitment, and levels of eotaxin-1, CXCL1/KC, IL-4, IL-5, IL-6, IL-13, and TNF; Increased serum levels of IL-10.
	Miranda et al. [41]	*A. muciniphila* BAA-835	
	Parrish et al. [42]	*A. muciniphila*	The presence of *A. muciniphila* in the microbiota, combined with fiber deprivation, led to stronger anti-commensal IgE coating and innate type 2 immune responses. This worsened food allergy symptoms in animal models of OVA and peanut allergy.
	Duan et al. [43]	*L. plantarum* JC7	Reduced plasma histamine levels, OVA-specific IgE serum levels, shift in Th1/Th2 immune response, and Treg/Th17 imbalance.
	Miranda et al. [44]	*Saccharomyces cerevisiae* UFMG A-905	Reduced tissue damage, myeloperoxidase activity levels, and IL-17 serum levels.
Shellfish	Fu et al. [45]	*Bacillus coagulans* 09.712	Improved gut barrier function; Suppression of the pro-inflammatory Th17 response.
	Fu et al. [46]	*L. casei* Zhang	Attenuated allergy symptoms and gut epithelial damage; Favoring a tolerogenic pattern through the activation of the NF-κB signaling pathway.
	Fu et al. [47]	*B. infantis*	Attenuated allergy symptoms; Induction of Tregs.

**Table 2:** Data from human studies related to food allergy prevention.

Study	Probiotic	Findings
Boyle et al. [48]	LGG to a pregnant mother	No reduction in the risk of eczema and food sensitization to eggs, peanuts, and cow’s milk in offspring
Dotterud et al. [49]	LGG, *L. acidophilus* La-5, and *B. animalis* subsp. lactis Bb-12 to a pregnant and lactating mother	Lower cumulative incidence of atopic dermatitis and no effect on atopic sensitization in offspring
Rautava et al. [50]	*L. rhamnosus* LPR and *B. longum* BL999 or *L. paracasei* ST11 and *B. longum* BL999 to pregnant and lactating mothers	Lower risk of eczema and no effect on atopic sensitization in offspring
Kuitunen et al. [51]	Probiotic mixture (lactobacilli, bifidobacteria, and propionibacteria) for pregnant mothers and their infants after birth	No difference in the cumulative incidence of allergic diseases and IgE sensitization at 5 years of life in offspring
Kalliomäki et al. [52]	LGG to the pregnant mother and their infants after birth	Lower incidence of early atopic diseases in high-risk children
Plummer et al. [53]	*B. infantis, B. lactis,* and *Streptococcus thermophilus* in very preterm newborns	No effect on the incidence of allergic diseases or atopic sensitization during the first 2 years of life
Morisset et al. [54]	Not hydrolyzed fermented formula containing heat-killed *B. breve* C50 and *Streptococcus thermophilus* 065 to infants at high risk of atopy	No effect on the incidence of CMA, a lower proportion of positive skin prick tests in cow’s milk, or a lower occurrence of allergy-like events in the first 2 years of life

**Table 3:** Data from human studies related to cow’s milk allergy treatment.

Study	Probiotic	Findings
Berni Canani et al. [61]	eHCF + LGG	eHCF + LGG induced a higher oral tolerance rate than eHCF alone or other special formulas in children with CMA
Guest et al. [62]	eHCF + LGG	eHCF + LGG induced a higher tolerance rate than eHCF alone or AAF in children with CMA
Berni Canani et al. [63]	eHCF + LGG	eHCF + LGG induced a higher oral tolerance rate and a lower incidence of atopic manifestations than eHCF alone in children with CMA with a follow-up of 36 months
Nocerino et al. [64]	eHCF + LGG	eHCF + LGG induced a higher oral tolerance rate and a lower incidence of atopic manifestations than other special formulas in children with CMA with a follow-up of 36 months
Guest et al. [65]	eHCF + LGG	eHCF + LGG is more effective than eHWF in both managing symptoms of CMA and preventing the occurrence of other atopic manifestations in children with CMA
Basturk et al. [66]	Milk-free diet + LGG	Milk-free diet + LGG improved symptoms such as bloody stools, diarrhea, restiveness, abdominal distension, mucous stools and vomiting in infants with CMA
Jing et al. [67]	Milk-free diet + *B. bifidum* TMC3115	Reduced allergic scores, improved anti-inflammatory responses, reduced serum IgE levels, increased serum IgG2 levels and improved gut microbiota in infants with CMA
Strisciuglio et al. [68]	Milk-free diet + Bifidobacteria	Decreased circulating naive and activated CD4+ T cells, as well as degranulating basophils, in infants with CMA
